# New-Onset Cancer in the HF Population: Epidemiology, Pathophysiology, and Clinical Management

**DOI:** 10.1007/s11897-021-00517-y

**Published:** 2021-06-28

**Authors:** Alessandra Cuomo, Francesca Paudice, Giovanni D’Angelo, Giovanni Perrotta, Antonio Carannante, Umberto Attanasio, Martina Iengo, Francesco Fiore, Carlo Gabriele Tocchetti, Valentina Mercurio, Flora Pirozzi

**Affiliations:** 1 Department of Translational Medical Sciences, Federico II University, Naples, Italy; 2 Interdepartmental Center of Clinical and Translational Sciences (CIRCET), Federico II University, Naples, Italy; 3 Interdepartmental Hypertension Research Center (CIRIAPA), Federico II University, Naples, Italy

**Keywords:** Heart failure, Cancer, Cardio-oncology, Cancer and heart failure risk factors, Epidemiology, Physiopathology

## Abstract

**Purpose of Review:**

Oncological treatments are known to induce cardiac toxicity, but the impact of new-onset cancer in patients with pre-existing HF remains unknown. This review focuses on the epidemiology, pathophysiological mechanisms, and clinical implications of HF patients who develop malignancies.

**Recent Findings:**

Novel findings suggest that HF and cancer, beside common risk factors, are deeply linked by shared pathophysiological mechanisms. In particular, HF itself may enhance carcinogenesis by producing pro-inflammatory cytokines, and it has been suggested that neurohormonal activation, commonly associated with the failing heart, might play a pivotal role in promoting neoplastic transformation.

**Summary:**

The risk of malignancies seems to be higher in HF patients compared to the general population, probably due to shared risk factors and common pathophysiological pathways. Additionally, management of these patients represents a challenge for clinicians, considering that the co-existence of these diseases significantly worsens patients’ prognosis and negatively affects therapeutic options for both diseases.

## Introduction

Cardiovascular disease (CVD) and cancer represent a burden for public health in industrialized countries. In recent years, outstanding progresses in the treatment of CVDs have led to a significant reduction of short-term mortality due to cardiovascular events, while the incidence of cardiac remodeling and consequent HF did not particularly change [1]. Hence, as the population ages, the incidence of both cancer and CVDs, especially heart failure (HF), increases.

In its earlier years, the field of cardio-oncology flourished with dealing with the cardiotoxic effects of antineoplastic therapies, aiming at improving cardiological care of cancer patients, considering that oncological treatments can induce cardiovascular issues that may ultimately lead to HF, even years after the completion of antineoplastic protocols [2••]. In recent years, cardio-oncology has substantially expanded, considering that new challenges rise from recent evidence that patients with HF have an increased risk of developing cancer, offering a new challenge to clinicians in terms of clinical and therapeutical management. In particular, this highlights the need to enhance the awareness on the relationship between cancer and HF and the mechanisms underlying it, in order to optimize the management and treatment of patients who present with both pathologies [3, 4].

**Fig. 1:**
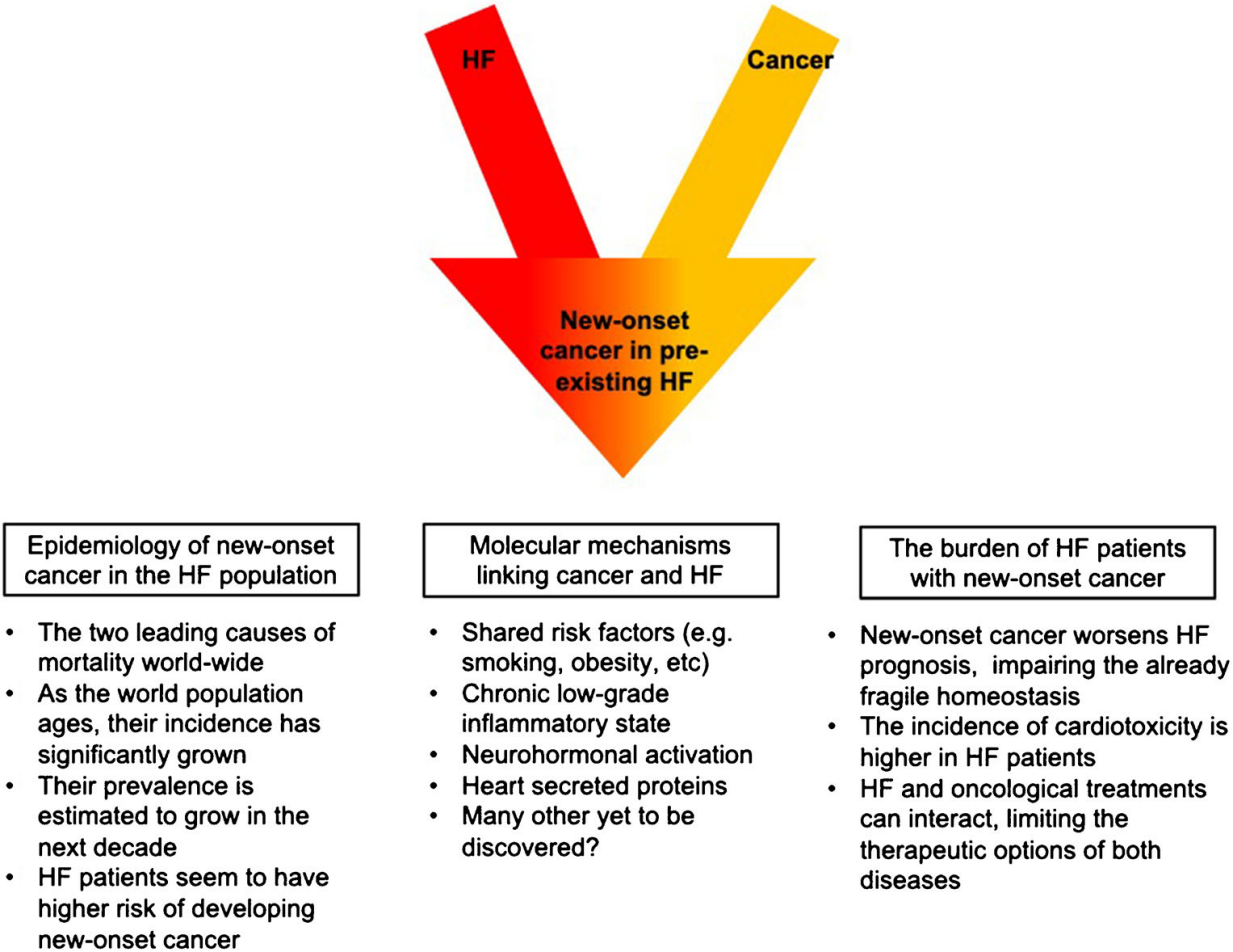
Summary of the epidemiology, pathophysiological mechanisms, and the clinical implications characterizing HF patients who develop malignancies. List of abbreviation: HF, heart failure

In this review, we discuss the most recent epidemiological and clinical evidence on new-onset cancer in the HF population, exploring the pathological mechanisms and clinical implications underlying the relationship between these two diseases and evidencing the main challenges for clinicians in optimizing cardiac therapy in this particular subset of patients, as summarized in Fig. 1.

## Epidemiology

Cancer and HF are leading causes of death worldwide, and their incidence increases with the progressive aging of the population [1]. Although these two conditions have been considered as distinct entities for long time, recent evidence indicates that they are deeply connected [5]. Moreover, along with the amelioration of CVDs management, the incidence of cardiovascular death has significantly decreased over the past decades, and long-term survivors to cardiovascular events may develop other age-related diseases, such as cancer, with consequent increase in non-cardiological deaths [6•, 7•].

Intriguingly, epidemiological and experimental studies show that patients with prior diagnosis of HF have a greater risk of developing new-onset cancer. For instance, in a case-control study, Hasin and coworkers compared cancer history in patient with newly diagnosed HF and patients without HF, detecting no association between cancer diagnosis and subsequent development of HF. When they explored the long-term risk of cancer in HF patients compared to general controls in a cohort study, after adjusting for major risk factors for both cancer and HF (such as diabetes, hypertension, and smoking), they found a 60% higher risk of developing cancer in patients with pre-existing HF. Moreover, they observed a 56% increased risk of death in patients with HF and incident cancer compared to HF patients who were not diagnosed with malignancies [8, 9•]. In another prospective cohort study, the aforementioned group investigated the incidence of tumors in patients who survived to a first myocardial infraction (MI). Interestingly, the authors discovered a 71% higher risk of succeeding cancer diagnosis in patients who develop HF after MI compared to those who did not develop it [10].

In another paper, Banke and colleagues examined the incidence of malignancies in a cluster of Danish patients with HF compared to the general population. After adjusting for shared risk factors, they found that HF patients presented greater risk of developing any type of cancer but prostate. In addition, they detected a rise in the mortality rates of HF patients with new cancer diagnosis compared to those who presented cancer without pre-existing HF, suggesting that the co-existence of these two conditions worsens patients’ prognosis [11•].

These data were also confirmed in a Japanese single-center study, in which researchers found an increased risk of stomach, lung, colon, breast, and prostate cancer in the HF cohort compared to controls, and, in addition, they reported a positive correlation between cancer incidence and brain natriuretic peptide (BNP) levels, although no correlation was found with left ventricular ejection fraction (LVEF) [12].

In a recent large retrospective study, the risk of cancer has been investigated in HF population compared to matched controls, using Danish nationwide administrative databases. The authors showed an increased incidence of all types of cancer, except melanoma, in HF group. However, after adjusting for common diseases (such as diabetes, COPD, chronic kidney disease, or prior MI present at baseline), the aforementioned risk is reduced, and it is even lower after further adjustment for administered medications (e.g., beta-blockers and inhibitors of the renin-angiotensin-aldosterone system). Intriguingly, these data suggest that cancer risk in HF population may be mostly driven by comorbidities and medications, other than HF itself [13].

Additionally, a prospective study explored cancer risk in HF population and found no association between these two pathologies [14]. This study involved a large sample for a long-term follow-up, but on the other hand, it included only male physician, and diagnosis of HF was not based on echocardiographic parameters but on patients’ self-report. Even though these data question the existence of a relationship between HF and cancer, more real-world studies are needed to understand whether these two diseases might be linked to one another [15].

Moreover, it is necessary to consider the possible presence of surveillance bias, acknowledging that HF patients undergo close follow-ups, with frequent clinical visits and diagnostic tests that could anticipate the diagnosis of malignancies compared to general population. However, cancer risk remained relevant even after eliminating malignancies detected in the first year [11•] and in the first 5 years of follow-up [8].

Importantly, symptoms such as dyspnea and fatigue can occur in both HF and cancer and may be misinterpreted as signs of HF worsening, procrastinating tumor diagnosis. On the contrary, common medications used for HF treatment could unmask malignancy that would have otherwise been silent for long, for example, gastrointestinal cancer may bleed earlier in patients who are treated with anti-platelet or anticoagulant drugs, commonly used for atrial fibrillation (AFib) or post-ischemic patients, both commonly associated with HF [3]. Indeed, in a recent manuscript, the authors evaluated the association between positive stool test for occult blood (fecal immunochemical test, FIT) and development of cardiovascular diseases, using the National Health Insurance database. After adjusting for sex, age, BMI, and common risk factors, they discovered that the FIT-positive group presented a higher risk of MI and ischemic stroke compared to patients with negative FIT. They also observed a 15% increase in mortality in the FIT-positive group even after taking out patients with colorectal cancers [16••].

Taken all together, these findings strongly suggest that the HF population presents an increased risk of developing malignancies that ultimately worsen prognosis.

## Pathophysiology of New-Onset Cancer in the HF Population

### Shared Risk Factors

The link between HF and cancer may be explained by the presence of overlapping risk factors, considering that a great number of risk factors are shared by malignancies and CVDs, especially HF, such as hypertension, diabetes, smoking, obesity, and aging [1, 4, 17].

In particular, it has been demonstrated that the risk of chronic conditions grows with aging, due to degenerative mechanisms, such as cellular senescence and oxidative stress [18••, 19].

Moreover, beside the well-known association between smoking and CVDs, there is strong evidence correlating tobacco usage and higher risk of malignancies, in particular lung cancer, while patients who stop or reduce smoking present lower incidence of tumors [20, 21]. Obesity is also a recognized risk factor for both CVD and cancer [22]. In particular, the risk of developing malignancies is increased in both obese and overweight patients, probably as a result of a state of chronic low-grade inflammation, which may conduct to DNA injury, mutations, and cancer induction [23, 24].

While the relationship between diabetes and cardiovascular disease has been largely investigated over the past decades [25], diabetes is also associated to increased risk of cancer development. For instance, in an Italian cohort study, diabetes patients had a greater risk of malignancies, especially patients on insulin therapy, compared to non-diabetic people [26].

### Neurohumoral System

Along with common risk factors, there are shared mechanisms linking both cancer and HF. In particular, the hyperactivation of the sympathetic nervous system (SNS), the renin-angiotensin-aldosterone system (RAAS), and the natriuretic peptide system all represent a hallmark of HF, and this neurohormonal activation is likely involved also in cancer development [4, 27].

It has been hypothesized that an excess of activity of β-adrenergic receptors (βARs) may conduct to tumor development through several molecular pathways (e.g., CREB, AP-1, and NF-kB) and induce cell proliferations via β-arrestin-1 signaling, giving resistance to apoptosis by inhibition of tumor suppressor gene p53, BAD (BCL2-associated death promoter), and anoikis, a type of programmed cell death that happens when cells detach from the extracellular matrix [28–31].

Moreover, not only are βARs involved in heart function, but also they are expressed in all types of cancer cells and also in cells from the tumor microenvironment [32]. In particular, it has been demonstrated that βARs activity stimulates tumor-associated macrophages to secrete prostaglandin E2, which increases the expression of vascular endothelial growth factor type C (VEGF-C), stimulating peri- and intra-tumoral lymph and blood vessels growth and promoting the subsequent tumor dissemination [33]. Finally, βARs hyperactivation enhances tumor spread and dissemination and also reduces natural killer cell activity, preventing them from removing transformed cells [34].

Interestingly, the angiotensin receptor 1 (AT1R) is also expressed by several types of tumor cells, corroborating the hypothesis that RAAS activation might be associated to the development, vascularization, and dissemination of malignancies [35]. On other hand, it has been observed that gene silencing and pharmacological blockade of the RAAS can reduce VEGF levels and subsequently tumor vascularization in animal models [36].

Surprisingly, clinical observations on the use of RAAS inhibitors have led to conflicting results [37, 38]. While data from the SOLVD and CHARM trials seem to show a positive correlation between ACE inhibitors or angiotensin receptor blockers (ARBs) and risk of cancer development, in a large meta-analysis, RAAS blockers showed a favorable effect on all cancer endpoints [39•].

In addition, angiotensin II seems to play a crucial role in VEGF-dependent angiogenesis [40], stimulating malignant cell proliferation and migration. Therefore, angiotensin antagonists might be considered possible therapeutic options in metastatic renal cell cancer and might also be added to FOLFIRINOX as part of the neoadjuvant protocol for the treatment of locally advanced pancreatic cancer [41, 42].

Atrial and brain natriuretic peptides may also play a role in cancer progression. For instance, their receptors are expressed by different kinds of tumor cells, and it has been observed that receptor A gene silencing attenuates tumor neo-angiogenesis and proliferation, but, conversely, ANP has also been shown to reduce tumor dissemination [27].

### Inflammation and Other Mediators

Another hypothesis, that may coexist with neurohormonal hyperactivation, suggests that both HF and cancer are characterized by the presence of low-grade inflammation [43]. While novel immune checkpoint inhibitors and CAR-T therapies are being developed in order to fight cancer [44••, 45, 46•], it is well-known that the inflammatory state plays a key role in starting and sustaining atherosclerosis, determining ischemic cardiovascular diseases and eventually HF. Moreover, microvascular endothelial inflammation may primarily determine a reduction of oxide nitric release, leading to cardiomyocytes hypertrophy and diastolic dysfunction that are characteristic of HF with preserved ejection fraction (HFpEF) [27, 47, 48].

Actually, it has been demonstrated that HF patients have higher circulating levels of pro-inflammatory cytokines (e.g., tumor necrosis factor-alpha, interleukin-1) [32]. In parallel, inflammation seems to stimulate cell proliferation, malignancy development, and progression [27]. Sustaining these hypotheses, the CANTOS trial (Canakinumab Anti-Inflammatory Thrombosis Outcome Study), proved that Canakinumab, the interleukin-1-β targeting antibody, decreases cardiovascular events in patients with story of MI and a moderate increase of C-reactive protein levels and has also shown a reduction of lung cancer incidence [49].

Furthermore, in a very interesting study, Meijers and coworkers investigated on the presence of causal relationship between HF and malignancy development. By inflicting large anterior MI, the authors induced HF in mice inclined to develop intestinal polyps and thus cancer. To exclude the influence of hemodynamic impairment, they transplanted the failing hearts into the cervical region of other mice with healthy hearts, and they found a greater tumor growth in the latter compared to non-transplanted mice. The authors also tested the hypothesis that the heart may secrete factors potentially associated with the increased cancer incidence and were able to identify five candidate proteins (SerpinA1, SerpinA3, fibronectin, ceruloplasmin, and paraoxonase 1). In order to further demonstrate their hypothesis, they also evaluated plasma from both HF patients and healthy controls enrolled in the PREVEND (Prevention of Renal and Vascular End-stage Disease) study and found that the same five proteins elevated in mice were increased in HF patients as well [50••].

Moreover, in another interesting study, the authors showed, in a mouse models of breast cancer, that the presence of MI intensified tumor growth through the epigenetic reprogramming of myeloid cells in hematopoietic reservoirs, activating monocytes and an immunosuppressive state. Additionally, they observed that in patient with early-stage breast cancer who developed cardiovascular events following cancer diagnosis, the risk of recurrence and death due to cancer was higher [51].

Finally, one recent study explored the connection between early heart remodeling, in absence of HF, and cancer growth. The authors investigated whether early stage of cardiac remodeling is sufficient to stimulate cancer development in a murine model of cardiac hypertrophy with transverse aortic constriction (TAC). Hence, they implanted breast and lung cancer models in these TAC mice and observed larger primary tumors and greater metastatic rates in TAC-operated mice compared to controls [52••]. Additionally, the authors analyzed data from an echocardiographic database and demonstrated that patients with moderate aortic stenosis and 40–60-year-old had a higher incidence of non-hematologic tumors compared to patients without aortic stenosis [52••], thus corroborating their experimental findings.

## Clinical Management of HF Patients Who Develop Cancer

Prognosis of both HF and cancer is poor and gets even worse when these two conditions coexist [1, 8]. Moreover, Banke and coworkers showed that HF patients present with higher risk of new cancer diagnosis, and after stratifying their study population for age, they also showed that HF patients with cancer had the same risk of death as patients 10 years older affected solely by HF (without cancer) [11•].

These data are partially ascribable to the independent death risk of each disease but also to the negative impact that one disorder has on the diagnosis, management, and treatment of the other, representing a challenge for clinicians. First of all, many symptoms of new-onset cancer, such as dyspnea and fatigue, frequently overlap with those of HF, and they may be considered as caused by worsening of HF, delaying tumor diagnosis [1].

Furthermore, cancer development may alter the vulnerable equilibrium of HF patients, amplifying chronic systemic inflammation and endothelial dysfunction and altering electrolytes and hormonal homeostasis [1]. Consequently, these patients are more prone to develop cardiotoxicities during or after antineoplastic therapies and have reduced chances to survive oncological surgery [53]. By limiting therapeutic choices, this may further worsen patients’ prognosis [54•, 55•, 56].

Moreover, we have to consider that both cancer and HF have a negative impact on patients’ mental health, often leading to depression and consequently growing patients’ mortality rate, seeing that the presence of depression is associated with increased mortality risk in both HF and cancer patients [57, 58].

It is likely that both neuronal changes per se and modifications in signaling and transmission underlie the clinical states of depression or cognitive changes in patients with concomitant HF and cancer. The most likely culprit is the chronic systemic inflammatory state present in both [4, 19], which is likely responsible for an enhanced level of oxidative stress, DNA damage, mitochondrial dysfunction, and synaptic modifications [59]. While there is data supporting a link between some chemotherapies and peripheral neuropathy (e.g., cisplatin), the issue of clinical states of depression/cognitive changes and them per se being a basis for autonomic dysfunction in this group of patients is far more complex and not demonstrated yet and deserves further investigation [60••].

Because of the complexity of this peculiar subset of patients, it is crucial to use an integrated clinical approach, involving not only cardiologists and oncologists but also other professionals, such as psychologists, pain therapists, and physical therapists [1]. Undoubtedly, a close cooperation between HF specialists and oncologists is required to establish the risk-benefit ratio of treatments and identify the best possible therapeutic path for the patients, avoiding the risk of undertreat either cancer or HF [54•].

The first step would be performing a baseline assessment [61••], including clinical history, physical examination, blood analysis, ECG, echocardiography, and any other test considered useful according to the clinical characteristic of each patient [58], for example, stress echocardiography or myocardial scintigraphy in HF patient with history of coronary artery disease, in order to identify residual myocardial ischemic areas [61••, 62••, 63••, 64••, 65••].

In this phase, before starting oncological treatments, it is fundamental to optimize cardiac therapies, acting also on modifiable risk factors—for example suggesting cessation of smoking, recommending lifestyle and diet changes, and enhancing diabetes therapy. Moreover, when needed, it is also crucial to treat valvular defects or residual myocardial ischemia. Unfortunately, optimization and up-titration of cardiological therapy may require weeks or even months, and this could represent a significant problem in the management of cancer patients, especially if this delays the beginning of oncologic treatments [1, 3, 66]. Furthermore, the correction of other conditions that may increase cardiotoxicity, such as metabolic and electrolyte alterations, is also important to prepare patients to better face future oncological treatments [54•, 55•, 56].

Another problem to be considered is that chemotherapy administration frequently requires the use of considerable quantity of fluids in order to reduce nephrotoxic effects [67]. This may be a concern in HF patients, in which fluid overload could cause significant complications, such as pulmonary and peripheral oedema. Therefore, in these patients, fluid infusion time should be prolonged, cumulative doses should be reduced, and diuretics could be selectively added, or diuretic medication doses increased the days within oncological treatment infusions [1, 66].

On the other hand, it is well-known that cancer diagnosis and follow-ups need the use of different types of imaging, often with iodinated contrast media, which are also associated with nephrotoxic effects. Patients might be infused with large quantities of fluids in order to avoid further kidney damages, but, as stated above, this could represent a burden for HF patients [68, 69].

It is critical to schedule regular follow-ups for HF patients during antineoplastic treatments, to early detect any sign of cardiotoxicity or serum alterations and quickly correct them, avoiding, when possible, the interruption of oncological treatments [1, 3].

An additional issue to take into account is the increased risk to develop QT prolongation and arrhythmia in cancer patients, such as AFib. Indeed, it has been demonstrated that the presence of cancer itself is associated with increased risk of developing AFib probably due to thoracic surgery, hypoxia, electrolytes, and metabolic alterations [70].

It is a common knowledge that AFib is a frequent comorbidity in HF patients, leading to higher death risk, and it represents also a possible cardiotoxic effect of antineoplastic treatments. For this reason, a frequent problem to be addressed in patients with HF and cancer is the choice of anticoagulant for thromboembolic event prevention [70, 71].

In the last years, direct oral anticoagulant has been preferred to warfarin because of their safety profile [72]. Nevertheless, for this subset of HF and cancer patients who have a greater risk of deep venous thrombosis, pulmonary embolism, and central venous thrombosis, long-term safety and efficacy have not yet been validated, and low molecular weight heparin represents the most used medicament. For this reason, it is very important for the cardio-oncology team to consider the thrombosis/bleeding risk for each individual patient and chose which anticoagulant to administer, if necessary, considering all possible drug interactions [3, 73].

Moreover, periodic clinical follow-ups should include electronic device interrogation (e.g., implantable pacemakers and defibrillators), especially in patients undergoing radiotherapy, which can also cause device malfunction [74].

The purpose of this integrated approach is to provide patients with best possible HF treatment to avoid being denied oncological therapies due to CVD, whereas it is not uncommon. Indeed, in a study with 5000 colorectal cancer patients, authors showed that those who are also affected by HF were less likely to receive adjuvant chemotherapy and had a worse 5-year prognosis, compared with non-HF cancer patients [75].

Additionally, in a population cohort study, Wang and colleagues evaluated the risk of cardiovascular long-term mortality in US 5-year survivors of adolescent and young adult cancer compared to the general population. They included 160,834 cancer patients, aged 15 to 39 years at diagnosis, from the SEER database. They detected that this subset of patients had 1.4 times increased risk of cardiovascular death than the US general population, with greatest risk for Hodgkin’s lymphoma patients and patients who were irradiated as part of their oncological protocols and the highest risk of cerebrovascular mortality for central nervous system tumor patients. They also noted that, even in patients who survived more than 30 years after cancer diagnosis, the risk of cardiovascular death remained markedly higher compared to controls [76]. Considering that patients included in the analysis were presumably treated with antineoplastic drugs for their malignancies, this data may partially be biased by the fact that oncological treatments are known to cause cardiotoxicity even years after the completion of anticancer therapies. Nevertheless, this study supports the hypothesis that, when dealing with fragile patients such as the HF population, cardio-oncologists need to be extremely aware of the possible cardiac complications associated with antineoplastic protocols.

It is clear that the lack of knowledge of the safety of oncological treatments in HF patients is a matter of concern, and trials should be designed to be as close as the real-world scenario, including the HF population. Recently, the SAFE-HEaRt trial has investigated whether patients with HF with mildly reduced ejection fraction might safely be treated with anti-HER2 drugs [77••].

Conversely, the risk of undertreating HF in cancer patients exists as well. Cancer alters HF patients’ homeostasis and can cause symptoms like vomiting and diarrhea, which can lead to electrolyte alterations, as well as endocrinological imbalance, with consequent possible reduction or suboptimization of HF therapy [3].

Furthermore, cancer patients are often excluded from therapies that would improve their life expectancy, such as heart transplantation or device implantation. For example, treatment with left ventricular assist devices should be considered in cancer patients with life expectancy of at least 2 years, or device implantation is denied when life expectancy is less than 1 year [66]. In these cases, instead, a close dialogue between cardiologists and oncologists would be essential in order to establish patient prognosis and life expectancy and to decide if patient is eligible for these treatments, since improving heart function, patient has better chance of continuing oncologic therapies [78].

Moreover, after heart transplantation, patients will need to take immunosuppressant drugs for a lifetime, and this represent a risk for further cancer development [66].

Finally, when the risk of cardiac events induced by oncological treatments is considered too high, oncologists should choose the antineoplastic protocol with less associated cardiotoxicity.

All things considered, it is clear that each HF patient who develops cancer has his/her own unique characteristics, and his/her clinical and therapeutical management should be personalized, in order to grant the best cardiological and oncological care.

## Conclusions

Recent evidence suggests that chronic diseases such as cancer and HF are closely related. In particular, not only do HF and cancer share the same risk factors, but also they present common pathophysiological mechanisms. Furthermore, it has been shown that the presence of one of these two diseases increases the risk of developing the other [4].

Intriguingly, the incidence of new-onset cancer in patients with pre-existing HF is higher than in the general population, and cancer patients have a greater risk of HF development, also due to cardiotoxic effects of antineoplastic treatments.

We believe that it would be useful to consider personalized surveillance programs to screen HF patients who are at higher risk for cancer development. For instance, further studies on factors secreted by the failing heart are required, to identify possible biomarkers to anticipate the diagnosis of new-onset cancer in this subset of patients. Indeed, novel serum factors may be use as cancer biomarkers helping to stratify cancer risk in HF patients [79]. Unfortunately, when these conditions coexist, patients’ mortality risk further increases, and it represents a challenge for clinicians, also considering that the treatment of one condition could hinder the one of the other.

In this scenario, a close dialogue between cardiologists and oncologists is essential to ameliorate clinical management and treatment of these patients. In particular, these specialists understand the need for optimized therapy in both HF and cancer patients, as well as the risk correlated the undertreatment, or even worse with the treatment interruption, of either one of these diseases.

A multidisciplinary approach with inclusion of other healthcare professionals such as psychologists and cardiac rehabilitation and palliative care specialists, when necessary, is then recommended [1, 78].

Further studies are needed to identify the limits for the administration of antineoplastic therapies in HF patients and optimal surveillance strategies for this unique subset of patients.
